# Une cause rare de dysphagie chez l'enfant: le bézoard

**DOI:** 10.11604/pamj.2014.18.109.4426

**Published:** 2014-06-03

**Authors:** Yousra El Boussaadni, Sahare El Mahjoubi, Aziza El Ouali, Wafaa Khannoussi, Noufissa Benajiba

**Affiliations:** 1 Université Mohamed Premier, CHU Mohamed VI, Département de Pédiatrie, Hopital Al Farabi, Oujda, Maroc; 2 Université Mohamed Premier, CHU Mohamed VI, Département de Gastroenterologie, Hopital Al Farabi, Oujda, Maroc

**Keywords:** Dysphagie, enfant, bézoard, endoscopie, dysphagia, child, bezoar, endoscopy

## Abstract

La dysphagie est un signe sémiologique peu fréquent chez l'enfant, dont l'oesophagite est souvent la cause. Les auteurs rapportent une cause rare de dysphagie chez une enfant de 3 ans et demi, ayant évolué 5 mois avant son admission et chez qui l'exploration para clinique a conclu à un bézoard. L'endoscopie a permis une extraction totale. À travers ce cas, nous essayons de rappeler aux praticiens les particularités cliniques de cette affection, ainsi que celles de la prise en charge et le suivi.

## Introduction

La dysphagie est un symptôme qui a une forte valeur sémiologique et qui ne doit pas être banalisé. De multiples étiologies peuvent être évoquées selon l’âge, les antécédents, le mode d’évolution ainsi que les données de l'examen clinique et les autres signes digestifs et extra digestifs associés. Nous rapportons une observation à propos d'une cause rare de dysphagie chez l'enfant [1, 2].

## Patient et observation

Il s'agit d'un garçon âgé de 3ans et 5mois, deuxième d'une fratrie de deux, sans antécédents pathologiques notables, admis dans notre formation pour la prise en charge d'une dysphagie aux solides évoluant depuis 5 mois associée à des épisodes de vomissements alimentaires et une notion d'amaigrissement chiffré à 3kg sans autres signes notamment pas de fièvre ni troubles de transit. L'examen clinique a noté une pâleur cutanéo-muqueuse et un retard staturo pondéral estimé à - 2DS pour le poids et la taille, sans signe de dénutrition ni de déshydratation. L'hémogramme a révélé une anémie hypochrome microcytaire (Hb à 6,9 g/dl d′hémoglobine, VGM à 48 fl et une CCMH à 29%).

**Figure 1:**
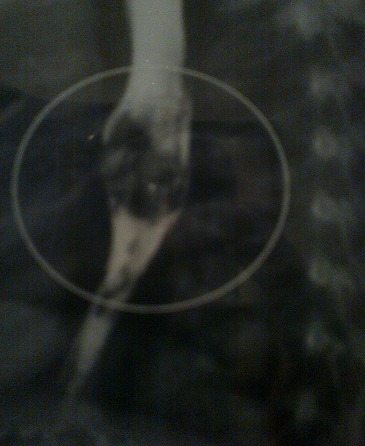
Un transit oesophagien objectivant une image lacunaire

**Figure 2:**
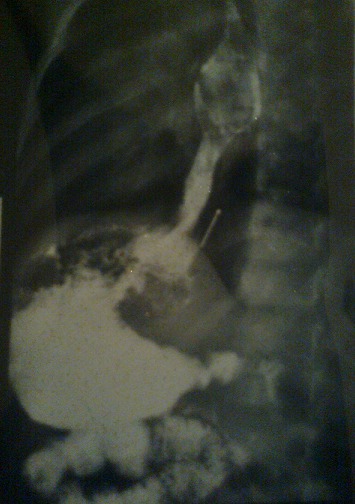
Un transit oesophagien mettant en évidence une sténose oesophagienne avec dilatation sus sténotique

Le transit oeso gastro duodénal réalisé a mis en évidence une sténose oesophagienne, une légère dilatation sus sténotique et une image lacunaire faisant évoquer un corps étranger ou un processus tumoral (Figure 1, Figure 2).

**Figure 3:**
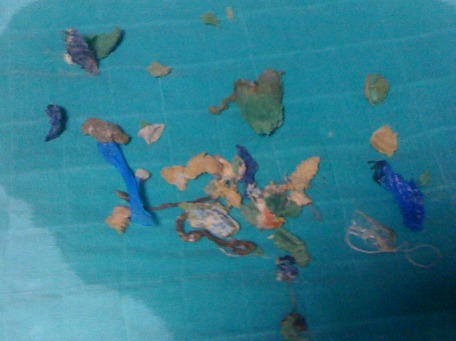
Multiples corps étrangers extraits par FOGD évoquant un bézoard

La fibroscopie a objectivé de multiples fragments de corps étrangers; mèches de cheveux, caoutchou, éponge (Figure 3). Le diagnostic de trichobézoard a été posé et une extraction endoscopique a été réalisée. L′évolution a été favorable avec une reprise normale de l'alimentation, disparition des vomissements et une bonne prise pondérale après un recul de six mois. Selon l′analyse psychopathologique, l'enfant présente un syndrome de Pica, et vu son âge la récidive du bézoard est très probable. En reprenant l'interrogatoire avec la famille, la maman a rapporté la notion d'une trichophagie et d'une géophagie.

## Discussion

La dysphagie reste un symptôme peu fréquent chez l'enfant, la fibroscopie ‘sogastroduodénale est l'examen clef. Les étiologies sont dominées par l'oesophagite, les troubles moteurs, alors que le corps étranger (bézoard) reste une étiologie rare mais non exceptionnelle [1]. Le bézoard est un terme issu du persan “panzehr”, ou l'arabe “badzehr” qui signifie antidote ou antipoison. Il désigne une affection rare, secondaire à l'accumulation de corps étrangers de diverses natures à l'intérieur du tube digestif et plus particulièrement au niveau de l′estomac [3, 4]. Le type du bézoard est déterminé par la nature des substances accumulées (le phytobézoard: résidus de débris végétaux, le lactobézoard composé de lait caillé, observé chez le nourrisson, le trichobézoard qui est formé d′un amas de poils ou de cheveux). D'autres bézoards ont été décrits après la prise de médicaments modifiant le comportement digestif: antiacides, choléstyramine appelés pharmacobézoard [5, 6].

Le trichobézoard est une affection rare chez l'enfant (0,15% des corps étrangers gastro-intestinaux), diagnostiquée le plus souvent à un âge avancé avec un pic entre 10 et 19 ans, cette affection est plus fréquente chez le sexe féminin (90% des cas), et il touche le grand enfant dans trois quarts des cas [6]. Le premier cas de bézoard a été décrit en 1779, et jusqu’à nos jours, il s′observe en particulier chez les sujets présentant des troubles neuropsychiatriques principalement le syndrome Pica ou chez ceux ayant subit une gastrectomie partielle. La particularité de notre observation est surtout la localisation oesophagienne souvent inhabituelle et méconnue devant la symptomatologie insidieuse [1].

La localisation gastro-intestinale est la plus fréquente, peut rester longtemps asymptomatique, ce qui explique le retard du diagnostic qui peut aller jusqu’à plusieurs années [7, 8]. Exceptionnellement ils peuvent se prolaber dans l′intestin grêle à travers le pylore et être source d'occlusion. La symptomatologie clinique est non spécifique souvent dominées par les troubles digestifs à type de: douleurs abdominales essentiellement de localisation épigastrique, des nausées, des vomissements alimentaires pouvant contenir des cheveux et parfois même des troubles du transit [6]. L'anorexie et l'amaigrissement peuvent être l’élément clinique majeur et évocateur. Ils ont été signalés dans notre observation au cours de l’évolution. L'interrogatoire doit préciser en plus des signes fonctionnels, la présence de facteurs favorisants: diabète, maladie systémique, dyspepsie idiopathique, un éventuel régime alimentaire riche en fibres, chirurgie de l'estomac, de l'oesophage et l'atrésie de l'oesophage. Les troubles de comportement alimentaire doivent être particulièrement recherchés type: titilomanie, trichotillomanie, trichophagie, car ils sont souvent niés par les parents [6, 7].

L'examen clinique, en dehors des complications, recherche une masse abdominale localisée le plus souvent au niveau de l'hypocondre gauche et/ou de l’épigastre. La découverte d'une plaque d'alopécie localisée, de caractère mécanique, est un signe d'orientation majeur et doit faire rechercher une trichophagie qui est le lien direct entre la trichotillomanie (souvent niés par les parents) et le trichobézoard. La présence d'une notion de satiété précoce est un signe d'orientation surtout si elle s'associe à une haleine fétide pouvant correspondre à la putréfaction des aliments. Ainsi la présence de facteurs favorisants et /ou l'association des signes révélateurs à une alopécie doit attirer l'attention du clinicien vers un éventuel trichobézoard [4, 9, 10]. Une fois le diagnostic du bézoard est évoqué cliniquement, il est nécessaire de le confirmer par des examens complémentaires. La fibroscopie oesogastroduodénale est la technique de choix dans les formes localisées gastriques et de petite taille, elle permet à la fois le diagnostic et l'extraction du corps étranger. D'autres examens radiologiques peuvent être demandés surtout devant un trichobézoard géant; l’échographie abdominale trouve toute son indication devant la palpation d'une masse abdominale chez l'enfant, l'aspect du bézoard est celui d'une masse hyperéchogène avec un cône d'ombre postérieur occupant la région épigastrique, en parallèle le transit ‘sogastroduodénal pose le diagnostic de bézoard gastrique dans la totalité des cas sous forme d'une image lacunaire intraluminale mobile, à bords convexes, et permet de préciser l'extension au niveau duodénojéjunal [4, 5, 11], comme c’était le cas dans notre observation. La TDM et l'IRM restent des techniques coûteuses qui ne sont pas indispensables pour le diagnostic du bézoard [4, 5].

Sur le plan thérapeutique, le choix de la technique est discuté selon les équipes en tenant compte de la taille du bézoard. L'usage de boissons abondantes associé à la prise d'accélérateurs du transit peut être proposé en cas de trichobézoard de petite taille. En cas d’échec, l'extraction endoscopique peut être tentée en s'aidant de rayon laser pour le fragmenter, ce qui a été le cas de notre patient chez qui la voie endoscopique a permis une extraction complète du bézoard [4, 11, 12]. Dans certains cas, le bézoard peut être fragmenté mécaniquement à l'aide d'une pince à biopsie et ensuite éliminé par lavage et aspiration avec un risque de perforation gastrique ou oesophagienne. Une dissolution peut être essayée au moyen de papaïne, d'acétylcystéine et de cellulase. Outre un traitement incomplet, ces méthodes exposent à un risque important de complications iatrogènes, en particulier oesophagiennes ou d'occlusion intestinale sur fragment de trichobézoard. De ce fait, le traitement est souvent chirurgical, en permettant en plus de l'extraction du bézoard et ses extensions, d'explorer tout le tube digestif, tout en sachant que la voie laparoscopique est récemment proposée comme alternative à la laparotomie [4, 13]. Malgré la description de certains cas compliqués par une occlusion du grêle, obstruction du pylore, perforation gastrique et quelques cas de pancréatite [4, 13, 14]. L’évolution reste souvent favorable surtout chez l'enfant, conditionnée aussi par une prise en charge psychiatrique se basant surtout sur la thérapie comportementale, éducation parentale et parfois même un traitement médical chez les patients présentant une trichophagie [3, 4, 15].

## Conclusion

Le trichobézoard est une affection rare qui touche neuf fois la fille plus que le garçon. Il est le plus souvent associé à des troubles de la conduite et du comportement alimentaire (trichotillomanie, trichophagie). Les signes cliniques sont digestifs souvent associés à des signes généraux. Le diagnostic fait appel à la radiologie notamment l’échographie, le TOGD et l'endoscopie. La voie endoscopique permet également de faire l'extraction selon la localisation alors que la chirurgie reste la méthode de choix en cas de bézoard de grande taille, d’échec ou en cas de complications.
